# Statistical optimization of process parameters for exopolysaccharide production by *Aureobasidium pullulans* using sweet potato based medium

**DOI:** 10.1007/s13205-015-0308-3

**Published:** 2015-06-02

**Authors:** Sethuraman Padmanaban, Nagarajan Balaji, Chandrasekaran Muthukumaran, Krishnamurthi Tamilarasan

**Affiliations:** Department of Biotechnology, Madha Engineering College, Kundrathur, Chennai, 600069 Tamilnadu India; Department of Industrial Biotechnology, Government College of Technology, Coimbatore, 641013 Tamilnadu India; Department of Chemical Engineering, School of Bioengineering, SRM University, Kattankulathur, Chennai, 603203 Tamilnadu India

**Keywords:** Sweet potato, Exopolysaccharide, Response surface methodology, Central composite design

## Abstract

Statistical experimental designs were applied to optimize the fermentation medium for exopolysaccharide (EPS) production. Plackett–Burman design was applied to identify the significance of seven medium variables, in which sweet potato and yeast extract were found to be the significant variables for EPS production. Central composite design was applied to evaluate the optimum condition of the selected variables. Maximum EPS production of 9.3 g/L was obtained with the predicted optimal level of sweet potato 10 %, yeast extract 0.75 %, 5.5 pH, and time 100 h. The determined (*R*^2^) value was 0.97, indicating a good fitted model for EPS production. Results of this study showed that sweet potato can be utilized as a low-cost effective substrate for pullulan production in submerged fermentation.

## Introduction

Biopolymers produced by a wide variety of microorganisms which are generally water soluble gums having novel and unique physical properties. Polysaccharides have found a wide range of applications in the food, pharmaceutical, and other industries. Typical industrial uses of EPS are as food coatings and packaging material due to its good film-forming properties similar to those of polyvinyl alcohol. Pullulan is used as an adhesive in the form of paste with water, as a construction material with fibers similar in strength and elasticity to those in nylon and as a bulking agent and stabilizer for tablets in the pharmaceutical industry (Deshpande et al. 1992). Many researchers have optimized the production conditions for exopolysaccharide (EPS) in submerged culture by *Fomes fomentarius* (Chen et al. 2008), *Tremella fuciformis* (Cho et al. 2006), *Pholiota squarrosa* (Wang et al. 2004), *Agrocybe cylindracea* (Kim et al. 2005), *Collybia maculate* (Lim et al. 2004), *Cordyceps jiangxiensis* (Xiao et al. 2004), *Cordyceps militaris* (Kim et al. 2003)*, Aureobasidium pullulans (*Moubasher and Wahsh 2014), and *Tremella mesenterica* (De Baets et al. 2002).

For a wide application, the cost of medium components is one of the main factors determining the economics of a process. In the literature, several agro-based products were utilized as low-cost substrate in the medium for economic production of pullulan through fermentation process (Srikanth et al. 2014; Sharmila et al. 2013a; Goksungur et al. 2011; Wu et al. 2009). In this study, sweet potato was used as an alternative low-cost carbon source for pullulan production.

Statistical experimental designs such as Plackett–Burman (BP) design and response surface methodology (RSM) are successfully employed to screen and optimize the process parameters in bioprocess field (Sharmila et al. 2013b). RSM, an experimental strategy for seeking the optimum conditions for a multivariable system, is a much more efficient technique for optimization (Alok et al. 2013; Aarthi and Karna 2012; Baskar and Renganathan 2012; Zhou et al. 2013; Mayur et al. 2013). Central composite design (CCD) is widely employed for bioprocess optimization studies and it can give information about the interaction between variables, provide information necessary for design and process optimization. The aim of the present work is to screen and optimize the process variables for EPS production from *A. pullulans* MTCC 2195 using statistical techniques.

## Materials and methods

### Microorganism and chemicals

*Aureobasidium pullulans* MTCC 2195 was obtained from Microbial Type Culture Collection and Gene Bank, Institute of Microbial Technology (IMTECH), India. Sucrose and yeast extract purchased from HiMedia Laboratories Pvt Ltd, (Mumbai, India). KH_2_PO_4_, MgSO_4_·7H_2_O, and NaCl were purchased from Qualigens fine chemicals. ZnSO_4_·7H_2_O and CuSO_4_·5H_2_O were procured from Loba Chemie. All the media components were of analytical grade, and solvents were purchased from Merck.

## Inoculum and substrate preparation

Inoculum was prepared by transferring a loopful of stock culture to the growth medium. Growth medium contains (w/v) sucrose 5 %, yeast extract 0.2 %, KH_2_PO_4_ 0.5 %, MgSO_4_.7H_2_O 0.02 %, and NaCl 0.1 %. The cultivation was performed at 35 °C for 3 days. Sweet potato was obtained from the local market in Chennai. It was ground into powder with a blender and passed through a sieve (80/100 mesh size) to remove large-sized particles, and the fine sweet potato powder was used for further studies.

### Fermentation conditions

*Aureobasidium pullulans* was inoculated in a production media containing (w/v) sweet potato 10 %, yeast extract 0.5 %, (NH_4_)_2_SO_4_ 0.4 %, NaNO_3_ 0.4 %, NaCl 0.2 %, KH_2_PO_4_ 0.2 %, MgSO_4_·7H_2_O 0.2 %, ZnSO_4_·7H_2_O 0.05 %, and CuSO_4_·5H_2_O 0.05 %, of pH 5.5. Fermentation at 35 °C for 120 h under shaking at 120 rpm with 2 % inoculum was performed. Five milliliters of the culture was taken at 120 h, and the culture was centrifuged at 14,000×*g*, 4 °C for 20 min. The culture supernatant was used for EPS precipitation.

### Estimation of exopolysaccharide

Supernatant fluid was mixed with three volumes of 95 % ethanol, stirred vigorously, and incubated at 4 °C for 24 h to precipitate the polysaccharide, which was separated by centrifugation at 14,000×*g* for 20 min and dried at 90 °C for 12 h (Wu et al. 2009). The EPS concentration was expressed as milligram per milliliter.

### Plackett–Burman design

Plackett–Burman design is used to screen the significant media components from large number of variables with minimum number of experiments (Plackett and Burman, 1946). This is a very economical factorial design with the run number a multiple of four and comprises two-level screening designs (Cupul et al. 2014). This design is extremely useful in screening importance of the factors affecting the production of polymer (Wang et al. 2014). This model describes no interaction among the factors that influences EPS production. Twelve experimental run was carried out to study the effect of seven medium components for EPS production. All the factors are prepared at two levels “−1” for low level and “+1” for high level. The seven factors, sweet potato, yeast extract, NH_4_SO_4_, NaNO_3_, NaCl, KH_2_PO_4_, and MgSO_4_, were studied on EPS production. ZnSO_4_·7H_2_O and CuSO_4_·5H_2_O were used as dummy variables. Table 1 shows the factors considered for investigation, and twelve experimental runs were carried out for EPS production. The fermentation was carried out for 120 h at 35 °C and 120 rpm.

**Table 1:** Plackett–Burman experimental design for screening of media components for EPS production

Std. order	Media components, (w/v) (%)							EPS (g/L)
	A	B	C	D	E	F	G	
1	15	0.5	0.6	0.3	0.1	0.1	0.3	7.2
2	15	1.0	0.3	0.6	0.1	0.1	0.1	7.5
3	5	1.0	0.6	0.3	0.3	0.1	0.1	4.0
4	15	0.5	0.6	0.6	0.1	0.3	0.1	6.8
5	15	1.0	0.3	0.6	0.3	0.1	0.3	8.0
6	15	1.0	0.6	0.3	0.3	0.3	0.1	9.0
7	5	1.0	0.6	0.6	0.1	0.3	0.3	4.0
8	5	0.5	0.6	0.6	0.3	0.1	0.3	2.0
9	5	0.5	0.3	0.6	0.3	0.3	0.1	3.0
10	15	0.5	0.3	0.3	0.3	0.3	0.3	6.8
11	5	1.0	0.3	0.3	0.1	0.3	0.3	4.5
12	5	0.5	0.3	0.3	0.1	0.1	0.1	3.0

### Central composite design

Central composite design developed by the Minitab 14 software was used to optimize the condition of the screened variables sweet potato, yeast extract, pH, and time. Other components of the medium were (w/v) (NH_4_)_2_SO_4_ 0.4 %, NaNO_3_ 0.4 %, NaCl 0.2 %, KH_2_PO_4_ 0.2 %, MgSO_4_.7H_2_O 0.2 %, ZnSO_4_·7H_2_O 0.05 %, and CuSO_4_·5H_2_O 0.05 % maintained as constant. Each factor in the design was studied at three levels. The minimum and maximum range of variables investigated and their values in actual and coded form are listed in Table 2. Experimental design includes 31 runs, and fermentation was carried out separately for each with replicates. The EPS concentration was taken as the dependent variable or response (Y). Regression analysis was performed on the data obtained. This resulted in an empirical model that related the response measured to the independent variables of the experiment. For any system, the model equation is represented as

1 $$Y = A_{ 0} + \varSigma \, A_{i} X_{i} + \varSigma \, A_{i} X_{i}^{ 2} + \varSigma \, A_{ij} X_{i} X_{j} ,$$

where *Y* is the predicted response, *A*_0_ the intercept, *A*_*i*_ the linear coefficient, and *A*_*ij*_ is the interaction coefficient. An analysis of variance (ANOVA) was performed, and three-dimensional response surface curves were plotted by Minitab 14 software to study the interaction among these factors.

**Table 2:** Statistical analysis of Plackett–Burman design on EPS production

Variables	Lower level (−1)	Higher level (+1)	Main effect	*t* value	*p* value	Confidence level (%)
Sweet potato, (X_1_)	5	15	4.13	16.35	<0.001	99.9
Yeast extract, (X_2_)	0.5	1.0	1.36	5.41	0.006	99.4
NH_4_SO_4_, (X_3_)	0.3	0.6	0.03	0.13	0.901	09.9
NaNO_3_, (X_4_)	0.3	0.6	−0.53	−2.11	0.102	89.8
NaCl, (X_5_)	0.1	0.3	−0.03	−0.13	0.901	09.9
KH_2_PO_4_, (X_6_)	0.1	0.3	0.40	1.58	0.189	81.1
MgSO_4_, (X_7_)	0.1	0.3	−0.13	−0.53	0.626	37.4

## Results and discussion

### Screening of media components for optimization process

Seven factors of media components were examined in the Plackett–Burman design experiments with twelve different trials, and maximum EPS production was obtained for trial number 6, while the lowest production was obtained for trial number 8. The regression coefficients, *t*-value, and confidence level are given in Table 2. The media components showed both positive and negative effects on EPS production. Statistical analysis (*t* values) demonstrated that sweet potato and yeast extract had significant positive influences on the EPS production with main effects of 4.13 and 1.36, respectively. The media components namely sweet potato (A) and yeast extract (B) were found to increase on EPS production at their high level. Whereas the media components namely (NH_4_)_2_SO_4_ (C), NaNO_3_ (D), NaCl (E), K_2_HPO_4_ (F), and MgSO_4_ (G) were found to decrease the EPS production at their higher level.

**Fig. 1:**
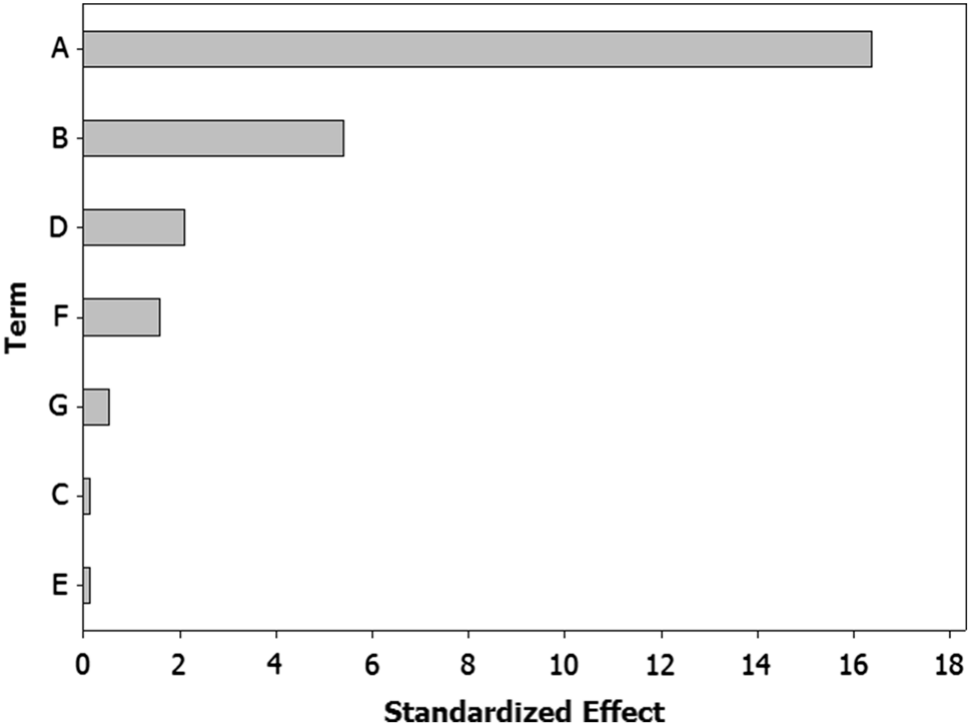
Pareto plot for Plackett–Burman parameter estimates for seven medium components. *A* Sweet potato, *B* yeast extract, *C* NH_4_SO_4_, *D* NaNO_3_, *E* NaCl, *F* KH_2_PO_4_, and *G* MgSO_4_

With the calculated p values at 90 % confidence level being considered to be significant factor, sweet potato (confidence level = 100 %) and yeast extract (confidence level = 99.4 %) were identified as the significant media components for EPS production. Hence, the main effect of media components was also studied graphically using Pareto chart as shown in Fig. 1. In this result, sweet potato has the highest confidence level of 100 %, indicating that it is the key factor for EPS production.

### Media optimization using central composite design

The final medium optimization and interaction amongst the screened factors were studied using CCD. All the experiments were carried out in replicates, and average EPS given in Table 3 was subjected to multiple linear regression analysis. The effect of sweet potato, yeast extract, pH, and time for EPS production was described in the form of second-order polynomial model in coded units (Eq. 2).

**Table 3:** CCD matrix of independent variables used in RSM with corresponding experimental and predicted values of EPS production

Std. order	A	B	C	D	EPS (g/L)	
					Experimental	Predicted
1	5	0.50	4.5	80	5.2	4.8
2	15	0.50	4.5	80	7.0	7.1
3	5	1.00	4.5	80	4.2	4.6
4	15	1.00	4.5	80	7.5	7.3
5	5	0.50	6.5	80	3.5	3.9
6	15	0.50	6.5	80	8.2	8.0
7	5	1.00	6.5	80	3.5	3.7
8	15	1.00	6.5	80	8.3	8.1
9	5	0.50	4.5	120	3.0	3.4
10	15	0.50	4.5	120	5.0	4.8
11	5	1.00	4.5	120	5.1	5.2
12	15	1.00	4.5	120	7.1	6.9
13	5	0.50	6.5	120	3.8	3.9
14	15	0.50	6.5	120	7.2	7.0
15	5	1.00	6.5	120	5.4	5.6
16	15	1.00	6.5	120	8.8	9.1
17	0	0.75	5.5	100	3.2	2.6
18	20	0.75	5.5	100	7.9	8.4
19	10	0.25	5.5	100	5.0	5.0
20	10	1.25	5.5	100	7.1	6.9
21	10	0.75	3.5	100	4.8	4.9
22	10	0.75	7.5	100	6.4	6.2
23	10	0.75	5.5	60	6.7	6.7
24	10	0.75	5.5	140	6.5	6.3
25	10	0.75	5.5	100	9.1	8.9
26	10	0.75	5.5	100	8.9	8.9
27	10	0.75	5.5	100	8.8	8.9
28	10	0.75	5.5	100	8.2	8.9
29	10	0.75	5.5	100	9.3	8.9
30	10	0.75	5.5	100	8.9	8.9
31	10	0.75	5.5	100	9.0	8.9

2 $$EPS\, ( {\text{g/L)}}\,=\,8.88 + 1.45 {\text{A }} + 0.46 {\text{B}} + 0.32 {\text{C}} {-}\,0.1 {\text{D}} {-}\,0.85 A^{2} {-}\,0.73 B^{2} {-}\,0.84 C^{2} {-}\,0.59 D^{2} +\,0.10 {\text{AB}} +\,0.45 {\text{AC}}{-}\,0.24 {\text{AD}}{-}\,0.02 {\text{BC}} +\,0.48 {\text{BD}} +\,0.33 {\text{CD}}.$$

The student’s *t* test and *F*-test were performed to determine the significance of the model. The residuals analysis was performed to validate the model at 95 % confidence level. In this model, the *R*^2^ value of 0.979 indicated that the response model can explain 97.9 % of the total variations. In general, a regression model having an *R*^2^ value higher than 0.9 is considered to have a very high correlation (Haaland 1989). The value of the adjusted determination coefficient (*R*_adj_^2^ = 96 %) was also high enough to indicate the significance of the model. The model fitted well with EPS production, and the optimal values from the model were justified (*p* < 0.001). The ANOVA results given in Table 4 indicate that the linear and square terms in second-order polynomial model (Eq. 2) were highly significant (*p* < 0.005) and adequate to represent the relationship between EPS production.

**Table 4:** Analysis of variance of second-order Polynomial model for effect of variable on EPS production

Sources	Coefficient	DF	SS	MS	*F* value	*p* value
*Model*	8.88	*14*	*119.772*	*8.5551*	*52.66*	<0.001*
*Linear*		*4*	*58.462*	*14.6154*	*89.96*	<0.001*
A:Sweet potato	1.45	1	50.460	50.4600	310.59	<0.001*
B:Yeast extract	0.46	1	5.227	5.2267	32.17	<0.001*
C:pH	0.32	1	2.535	2.5350	15.60	0.001*
D:Time	−0.1	1	0.240	0.2400	1.48	0.242
*Square*		*4*	*51.372*	*12.8431*	*79.05*	<0.001*
Sweet potato*sweet potato	−0.85	1	21.097	21.0967	129.86	<0.001*
Yeast extract*Yeast extract	−0.73	1	15.403	15.4031	94.81	<0.001*
pH*pH	−0.84	1	20.487	20.4872	126.10	<0.001*
Time*time	−0.59	1	10.172	10.1723	62.61	<0.001*
*2*-*Way interaction*		*6*	*9.938*	*1.6563*	*10.19*	<0.001*
Sweet potato*yeast extract	0.1	1	0.160	0.1600	0.98	0.336
Sweet potato*pH	0.45	1	3.240	3.2400	19.94	<0.001*
Sweet potato*time	−0.24	1	0.902	0.9025	5.56	0.032*
Yeast extract*pH	−0.02	1	0.010	0.0100	0.06	0.807
Yeast extract*time	0.48	1	3.802	3.8025	23.41	<0.001*
pH*time	0.33	1	1.823	1.8225	11.22	0.004*
*Error*		*16*	*2.599*	*0.1625*		
Lack-of-fit		10	1.891	0.1891	1.60	0.292
Pure error		6	0.709	0.1181		
Total		30	122.371			
*R* ^2^	0.97					
*R* ^2^_(adj)_	0.96					

* Significant model terms (*p* < 0.005)

**Fig. 2:**
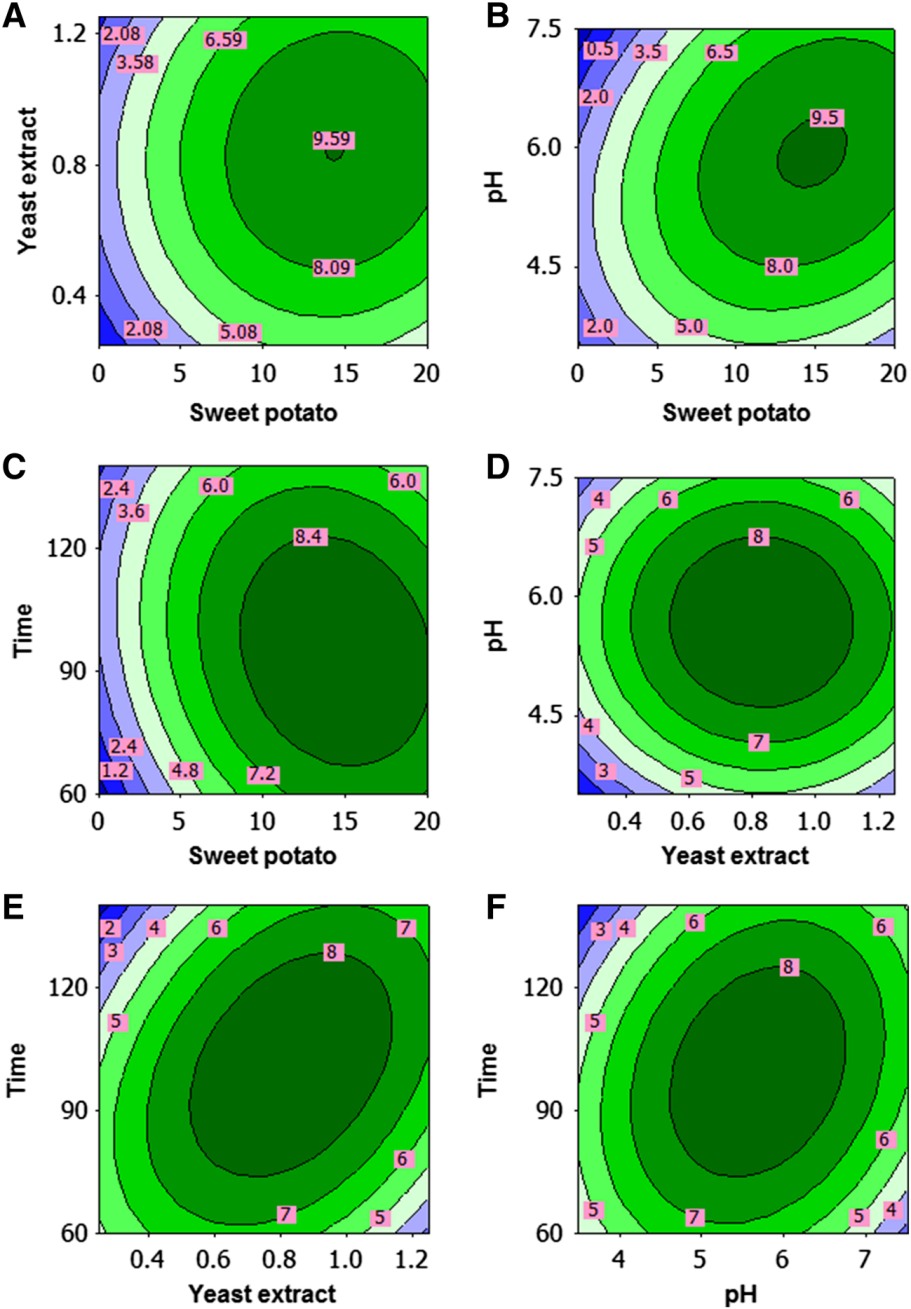
Contour plots showing the combined effect of the medium variables (sweet potato, yeast extract, pH, and time) on EPS production by *Aureobasidium pullulans*

The 3D response surface or contour plots were employed to determine the interaction of the fermentation conditions and the optimum levels for EPS production. The shape of the contour plot is used to identify the interaction of variables. Strong interaction exists if contour lines are elliptical in shape, and no interaction is observed if contour lines are circular. The mutual effect of sweet potato and yeast extract is shown in Fig. 2a. Maximum EPS production was obtained at the middle level of the variables and there was no interaction between the variables as contour lines are circular in shape. Figure 2b shows the combined effect of sweet potato and pH on the response, and a significant interaction exists between sweet potato and pH. The amylase enzyme synthesized by *A. pullulans* strain can degrade the sweet potato starch into simple carbohydrate molecules and be utilized for pullulan production as reported in previous studies (Manitchotpisit et al. 2011; Saha and Bothast 1993). As can be observed from Fig. 2c, EPS level increases as the time and sweet potato concentration increase till an optimal point is reached and EPS production decreases with further increase of time. Figure 2d shows that high and low level of yeast extract and pH have no significant effect on the EPS production and increased production was observed at middle level of yeast extract with pH. The interaction effect of yeast extract and time on EPS production is shown in Fig. 2e, while other factors were fixed as a constant. It was observed that the EPS production was reduced at low and high level, whereas increases towards middle level of yeast extract and time. Yeast extract in the production medium is essential nutrient to switch the morphological character (mycelial growth) of *A. pullulans* for pullulan synthesis. Figure 2f shows that strong interaction was observed between pH and time on EPS production as the contour is elliptical and maximum level was observed at middle level of pH and time. In other reports, optimal conditions for polymer production were obtained at an initial pH of 5.0 (Vijayendra et al. 2001), 6.5 (Roukas and Biliaderis 1995), and 7.5 (Auer and Seviour 1990). The different optimal initial pH values reported in the literature may be due to the different strains, compositions of fermentation media, and culture conditions used in those studies. Maximum EPS of 9.3 g/L obtained in this study has good agreement with the results reported by Moubasher and Wahsh (2014). In contrast to our results, maximum pullulan yield was obtained with sweet potato starch and hydrolysed potato starch waste as reported earlier (Goksungur et al. 2011; Wu et al. 2009). The variation in pullulan production may be due to type of *A. pullulans* strain, medium components, and process conditions.

## Conclusion

Statistical optimization was confirmed to be a powerful method for the optimization of the EPS production by *A. pullulans* MTCC 2195. The medium components, sweet potato and yeast extract, were screened to be the most significant components that influence the EPS production by PB experiment. CCD was proposed to study the interaction effects of fermentation condition. Maximum EPS production 9.3 g/L was obtained using the optimized condition of sweet potato 10 (%), yeast extract 0.75 (%), 5.5 pH, and time 100 h. Results of this showed that sweet potato may be used as an alternate carbon source for economical production of pullulan biopolymer.
